# Ursolic Acid Potentializes Conventional Therapy in Experimental Leishmaniasis

**DOI:** 10.3390/pathogens9100855

**Published:** 2020-10-20

**Authors:** Jéssica Adriana Jesus, Thays Nicolli Fragoso da Silva, Eduardo Seiji Yamamoto, João Henrique G. Lago, Márcia Dalastra Laurenti, Luiz Felipe Domingues Passero

**Affiliations:** 1 Laboratory of Pathology of Infectious Diseases (LIM50), Department of Pathology, Medical School of São Paulo University, Av. Dr. Arnaldo, 455, São Paulo, SP 01246-903, Brazil; jessicaa@usp.br (J.A.J.); thays.nicolli@gmail.com (T.N.F.d.S.); seijieduardo@me.com (E.S.Y.); mdlauren@usp.br (M.D.L.); 2 Center of Natural and Human Sciences, Federal University of ABC (UFABC), Avenida dos Estados 5001, Santo André, SP 09210-580, Brazil; joaohglago@gmail.com; 3 Institute of Biosciences, São Paulo State University (UNESP), Praça Infante Dom Henrique, s/n, São Vicente, SP 11330-900, Brazil; 4 Institute for Advanced Studies of Ocean, São Paulo State University (UNESP), Rua João Francisco Bensdorp, 1178, São Vicente, SP 11350-011, Brazil

**Keywords:** leishmaniasis, cutaneous leishmaniasis, visceral leishmaniasis, glucantime, amphotericin B, therapy

## Abstract

Ursolic acid (UA) is a triterpene with a broad array of pharmacological activities. In leishmaniasis, UA killed different species of parasites, and it was active in the experimental model of cutaneous and visceral leishmaniasis. Thus, the objective of this work was to study the therapeutic efficacy of the conventional drugs amphotericin B (AmB) or glucantime (Glu) combined with UA in experimental visceral and cutaneous leishmaniasis, respectively. *L. (L.) infantum*-infected hamsters were treated with AmB alone or combined with UA. *L. (L.) amazonensis-*infected BALB/c mice were treated with Glu alone or combined with UA. Animals were treated for 15 consecutive days by intraperitoneal or intralesional routes. Following one week after the last dose, the tissue parasitism and cellular immune responses were analyzed. Hamsters treated with 0.2 and 1.0 mg/kg of AmB plus 1.0 mg/kg of UA showed low hepatic and splenic parasitisms; however, AmB given as monotherapy did not reduce the number of viable parasites in the spleen of treated animals. In cutaneous leishmaniasis, Glu given as monotherapy was inactive at 2.0 mg/kg, showed mild activity at 10.0 mg/kg, and at 50.0 mg/kg was highly active at eliminating parasites in the skin. When animals were treated with Glu plus UA, higher leishmanicidal activity was observed in comparison to all groups treated with monotherapy schemes, and such activity was related to lesion improvement and upregulation of IFN-γ production. Altogether, data suggest that the association of drugs for the treatment of leishmaniasis can increase the efficiency of the treatment and decrease the toxicity associated to the conventional drugs.

## 1. Introduction

Leishmaniasis is a parasitic disease that is found mainly in tropical and subtropical areas, and has a prevalence of approximately 12 million cases; over 350 million people live in areas at risk of transmission [1]. It is estimated that 0.7 to 1 million new cases and about 26,000 to 65,000 deaths occur annually [1]. Considering the main clinical forms, approximately 58,000 cases of visceral and 220,000 cases of cutaneous leishmaniases are officially reported per year [2].

Depending on the infecting species, several clinical forms of leishmaniasis can be characterized, such as visceral or cutaneous forms. Visceral leishmaniasis (VL) or kala-azar is a severe and potentially fatal systemic disease [3]. Cutaneous leishmaniasis (CL), on the other hand, can manifest as a single skin lesion at the vector bite site to less common clinical forms with multiple nodules throughout the body, such as anergic cutaneous diffuse leishmaniasis that can cause deformities and sequelae [3]. The evolution and occurrence of less common or recurrent clinical forms depend on the host’s general state of health, host genetics, parasite species, virulence, as well as parasite-host interactions [4].

The therapeutic options for visceral and cutaneous leishmaniasis are focused on two major drugs, the pentavalent antimonials and amphotericin B [5]. In addition, other alternative drugs or formulations have been employed in the treatment of leishmaniasis, as is the case of the highly active drug Ambisome^®^, a liposomal drug containing amphotericin B, which has been used to minimize all side-effects caused by amphotericin B; however, the high costs related to the treatment is a major drawback in low income countries, such as East Africa and Brazil [6]. More recently, the antineoplastic miltefosine, the only available oral antileishmanial drug, has been used in some countries, but over a decade of use, its effectiveness has decreased significantly [7]. Of note, all conventional drugs induce local and systemic side-effects in patients, and reports about the emergence of parasite resistance have been constantly published, limiting their use [8,9].

In infectious diseases, as well as leishmaniasis, monotherapy has been associated with the emergence of microbial drug resistance [10], and thus the association of drugs represents a promising approach to eliminate intracellular parasites faster and at the same time avoid the emergence of resistance in *Leishmania* species. [10,11]. Association between drugs has been considered a consensus among specialists for several reasons [6]. Combination of drugs from different chemical classes may increase the effectiveness of therapy, reduce the dose and time of treatment that has a direct effect in the toxicity, and consequently patient tolerance and compliance increases during the treatment [6]. Combined therapy can delay the onset of resistance and increase the shelf life of different drugs, as has been observed in the treatment of some infectious diseases, such as malaria, tuberculosis, and HIV [10,12,13].

Clinical studies have investigated combination therapy, for example, using miltefosine (Mil) with other antileishmanial drugs in the treatment of VL [7,14]. Two studies conducted in India showed that the association of Mil with a single injection of liposomal AmB (5.0 mg/kg) reduced the treatment time with Mil, which alone would take about 28 days to cure patients with visceral leishmaniasis, whereas patients under combined therapy were cured after seven days [15,16]. In CL, previous studies have indicated that Glu associated with other drugs was more effective at eliminating parasites compared with Glu alone [17]. In this specific case, Glu combined with paromomycin or Mil synergistically reduced skin parasitism [17].

Due to the serious side-effects of drugs commonly used in the chemotherapy of leishmaniasis and the emergence of parasitic resistance, it is necessary to look for new therapeutic targets and strategies, which require shorter administration cycles, are more effective, and less toxic to patients [10]. Special metabolites from plants represent an interesting alternative in the search for new bioactive compounds, since different molecules have already been described with leishmanicidal activity [18], such as ursolic acid (UA).

UA is a natural pentacyclic triterpene isolated from several medicinal plants such as *Baccharis uncinella, Rosemarinus officinalis,* and *Radix actinidiae*; it has anti-inflammatory [19], anti-cancer [20], and anti-microbial activities [20,21,22,23,24,25]. In *L. (L.) amazonensis,* UA induced morphological, physiological, and biochemical alterations that resembled programmed cell death; additionally, BALB/c mice treated with UA by the intralesional route improved cutaneous lesions and showed lower tissue parasites than non-treated infected mice [26]. Golden hamsters infected with *L. (L.) infantum* under treatment with UA by the intraperitoneal route showed fewer parasites in the spleen and liver; moreover, animals under UA treatment produced high amounts of inducible nitric oxide synthase in the spleen, suggesting an enhancement of immune response in treated animals. Furthermore, UA was safe to be administered to BALB/c and golden hamsters since no histological and blood biochemical changes were detected in UA-treated animals [21,26]. Thus, this compound has been proven to be safe and more importantly, effective in both cutaneous and visceral experimental leishmaniasis, indicating that it may be considered an important target to develop new drugs directed to the treatment of leishmaniasis.

In order to avoid the emergence of parasites resistance, increase the efficacy, and decrease the total amount of classical drugs used in the therapy, the aim of this study is to investigate if the association between UA with amphotericin B or glucantime can improve the efficacy of the treatment for leishmaniasis.

## 2. Results

### 2.1. Chemical Analysis of Ursolic Acid

^1^H and ^13^C NMR data were compared with those previously reported in the literature [27,28,29]. These results, in association with elemental analysis data, indicated that UA exhibited 100% of purity.

### 2.2. Evaluation of the Therapeutic Potential of Drug Association in Experimental Visceral Leishmaniasis

**Figure 1:**
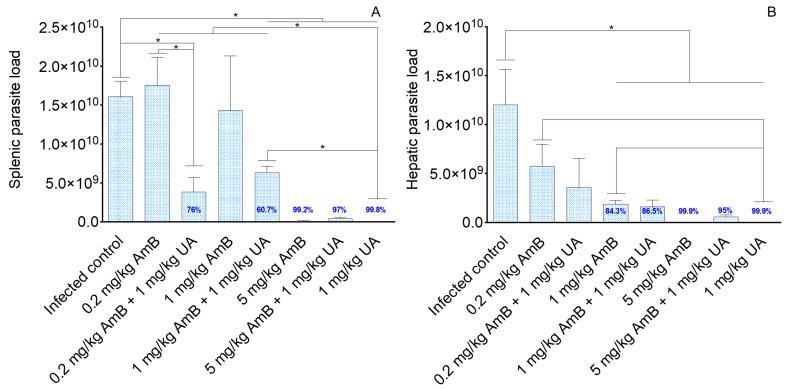
Golden hamsters were infected intraperitoneally with 2 × 10^7^ promastigote forms of *L. (L.) infantum*. Four weeks after infection, the animals were intralesionally treated once daily for 15 days with amphotericin B (AmB), ursolic acid (UA), or AmB plus UA. One week after the last injection, the number of viable parasites was estimated in the spleen (**A**) and liver (**B**) by limiting-dilution assay * *p* < 0.05.

Hamsters infected with *L. (L.) infantum* and treated with 0.2 and 1.0 mg/kg AmB did not reduce splenic parasitism in comparison to the infected control but when 1.0 mg/kg of UA was added to these doses of AmB, significant reductions in tissue parasitism (76 and 60.7, respectively, *p* < 0.05) were observed (Figure 1A). Animals treated with 5.0 mg/kg AmB alone or associated to UA (5.0 mg/kg AmB plus UA) showed 99.2 and 97.0% of reduction in the splenic parasitism when compared to the infected control group, respectively (Figure 1A); however, between these same groups, no significative differences of therapeutic activity were observed (*p* > 0.05). Additionally, animals treated with 1.0 mg/kg of UA alone showed significant reduction in splenic parasitism (99.8%) compared to groups treated with monotherapy (0.2 or 1.0 mg/kg of AmB) and combined therapy (0.2 mg/kg AmB plus UA; 1.0 mg/kg of AmB plus UA) as well as infected control (*p* < 0.05).

In the liver (Figure 1B), it was observed that animals treated with 0.2 mg/kg of AmB did not show significant reduction in hepatic parasitism (*p* > 0.05) in comparison to infected control; in contrast, animals treated with 1.0 and 5.0 mg/kg AmB alone or in association with UA (1.0 mg/kg) presented significantly less tissue parasites than the infected control group (*p* < 0.05). Animals treated with 1.0 mg/kg of UA presented significant reduction (*p* < 0.05) of the liver parasitism by 99.9% and comparatively it was more effective at eliminating parasites than 0.2 and 1 mg/kg of AmB alone (*p* < 0.05), as shown in Figure 1B.

### 2.3. Analysis of Cellular Immune Response in Visceral Leishmaniasis

**Figure 2:**
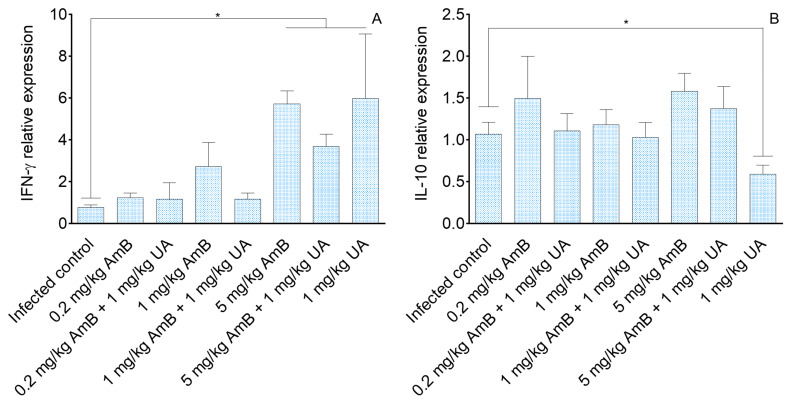
IFN-γ (**A**) and IL-10 (**B**) relative gene expression in the spleen of infected golden hamsters treated with amphotericin B (AmB), ursolic acid (UA), or AmB plus UA. Relative gene expression was estimated by quantitative PCR. The expression levels of genes of interest were normalized to β-actin. * *p* < 0.05 indicates statistical significance.

Analysis of cellular immune response in the spleen of *L. (L.) infantum*-infected hamsters showed that only animals treated with 1.0 mg/kg of UA, 5.0 mg/kg of AmB, and the therapeutic combination of 5.0 mg/kg AmB + 1.0 mg/kg UA expressed higher levels of IFN-γ compared to the infected group (Figure 2A, *p* < 0.05). Differences in the expression of IFN-γ were not observed between groups treated with mono and combined therapies.

The expression of IL-10 (Figure 2B) was similar among groups, except in animals treated with 1.0 mg/kg of UA that presented significant reduction in IL-10 expression, in comparison to the infected control (*p* < 0.05).

### 2.4. Evaluation of the Therapeutic Potential of Drug Association in Experimental Cutaneous Leishmaniasis

**Figure 3:**
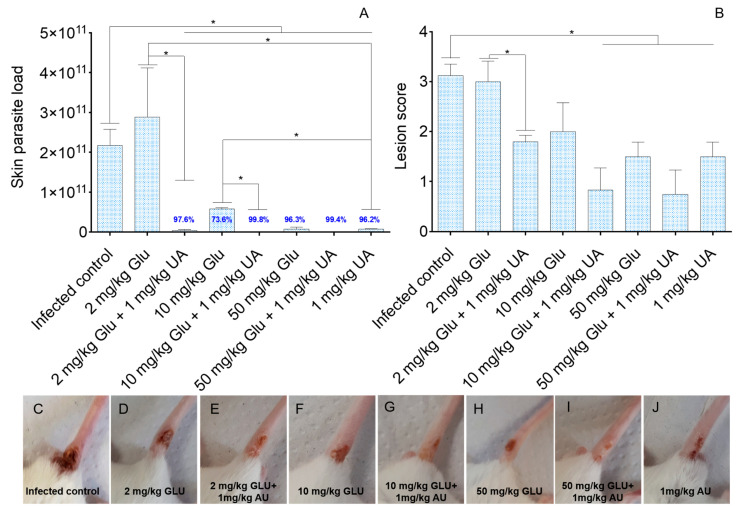
BALB/c mice were infected into the base of the tail with 10^6^ promastigote forms of *L. (L.) amazonensis.* Four weeks after the infection, the animals were intralesionally treated once daily for 15 days with Glucantime (Glu), ursolic acid (UA), or Glu associated with UA. Skin parasite load, quantified by limiting-dilution assay (**A**), was analyzed in the eighth week post-infection. Lesions were graded as 0 (no lesion), 1 (small infiltrative plaque), 2 (medium infiltrative plaques), 3 (large infiltrative plaques), and 4 (large ulcerated and necrotic plaques) according to the morphology of the lesions (**B**). Macroscopic images of the skin lesions from the infected control and treated groups (**C**–**J**). * *p* < 0.05 indicates statistical significance.

Infected BALB/c mice treated with 2.0 mg/kg of GLU showed no reduction in skin parasitism in comparison to the infected control (Figure 3A, *p* > 0.05). In comparison to the infected control, this treatment did not change the morphology of the skin lesions that were characterized by ulcerated and necrotic plates (Figure 3B–D, *p* > 0.05). On the other hand, when 2.0 mg/kg of Glu was associated with UA, a significant decrease in skin parasitism was observed, compared to the infected control and to animals treated with 2.0 mg/kg of Glu alone (Figure 3A, *p* < 0.05). Additionally, animals treated with 2.0 mg/kg of Glu plus UA presented skin lesions characterized by plaques of medium size in the base of the tail, which was smaller in comparison to the infected control and animals treated with 2.0 mg/kg of Glu alone (Figure 3B,E, *p* < 0.05). Animals treated with 10.0 mg/kg of Glu alone showed significant reduction in tissue parasitism (73.6%) compared to the infected control (*p* < 0.05), and a significative improvement in skin lesions was verified, characterized as infiltrative plaques of medium size (Figure 3B,F). When 10.0 mg/kg of Glu was associated with UA, significant reductions in skin parasitism were observed in comparison to skin parasitism from the infected control and animals treated with 10.0 mg/kg of Glu alone (*p* < 0.05). Both monotherapy and combined treatments improved the skin lesions morphology in comparison with the infected control (Figure 3B,F,G; *p* < 0.05). Animals treated with 50.0 mg/kg of Glu showed significant reduction in tissue parasitism of 96.3% in comparison with the infected control (Figure 3A), and when UA was associated with 50.0 mg/kg of Glu, the parasitism decreased by 99.4% compared to the infected control group (*p* < 0.05). Although less parasites were observed in the combined therapy (50.0 mg/kg of Glu plus UA), this difference was not significant (*p* > 0.05). Similarly, animals treated with 50.0 mg/kg of Glu alone or combined with UA showed infiltrative plaques of small size compared to the infected control (Figure 3B,H,I; *p* < 0.05). Comparatively, mono (50.0 mg/kg of Glu) or combined therapies (50.0 mg/kg of Glu plus UA) did not improve the overall aspect of skin lesions (*p* > 0.05). Animals treated with UA alone showed reduced skin parasitism in comparison with the infected control and animals treated with 2.0 and 10.0 mg of Glu (*p* < 0.05). Morphologically small infiltrative plaques were identified in the base of tail from animals treated with UA as monotherapy (Figure 3B,J; *p* < 0.05).

### 2.5. Analysis of Cellular Immune Response in Experimental Cutaneous Leishmaniasis

**Figure 4:**
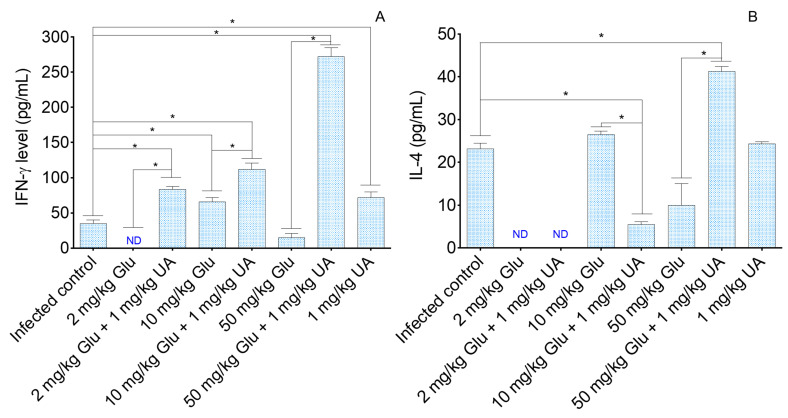
Mononuclear cells from the lymph nodes of treated and control BALB/c mice were isolated and cultured in 72 h under specific stimulation with the whole antigen of *L. (L.) amazonensis* (AgT), following the levels of IL-4 (**A**) and IFN-γ cytokines (**B**) were quantified by ELISA. * *p* < 0.05 indicates statistical significance.

Lymph nodes cells were stimulated with AgT from *L. (L.) amazonensis* and the amount of IFN-γ and IL-4 was quantified by ELISA. The treatments, performed as mono or combined therapies, enhanced the ability of lymph node cells to produce IFN-γ, except in the group treated with 2.0 and 50.0 mg/kg of Glu (Figure 4A). Additionally, it was verified that lymph node cells from animals treated with the combined therapy produced high levels of IFN-γ compared to the lymph node cells from animals treated with Glu alone (*p* < 0.05).

In comparison with the infected control (Figure 4B), IL-4 levels were increased in the group treated with the association between 50.0 mg/kg of Glu and UA (*p* < 0.05) and low levels of IL-4 were detected in animals treated with the association between 10.0 mg/kg Glu and UA (*p* < 0.05). Comparatively, it was observed that the association of 10.0 mg/kg of Glu with UA reduced the ability of lymph node cells to produce IL-4, and in contrast, the association of 50.0 mg/kg of Glu enhanced the ability of lymph node cells to produce IL-4, as illustrated in Figure 4B (*p* < 0.05).

## 3. Discussion

The association of drugs offers relevant benefits in the treatment of infectious diseases and presents advantages over monotherapy. Clinical studies have indicated that combined treatments can have three major consequences: (1) the clinical effects may be the sum of the effect of each drug alone (additive effect); (2) the effect of adding a second drug may exceed the individual effect of each drug alone (synergistic effect), or (3) the interaction between two drugs have opposite desired actions (antagonistic effect) [10]. As a consequence of additive or synergistic effects, patients can be treated with lesser amount of drugs in comparison to monotherapy [10], which, in fact can increase tolerability of the treatment, reduce side-effects of drugs, and decreases the costs of hospitalization. Additionally, combined therapy has been considered an important strategy in increasing the efficacy of drugs in infectious diseases, and prevents microbial resistance [30]. In the present study, it was verified that the association of UA with classical drugs used in the therapy of leishmaniasis was able to reduce parasite survival and the total amount of amphotericin B or glucantime injected in the experimental animals.

In experimental VL, it was verified that 0.2 mg/kg of AmB presented efficacy at reducing parasites in the spleen and liver of infected hamsters; and the dose of 1.0 mg/kg of AmB reduced the number of amastigote forms only in the liver. By adding UA to the therapy, significant reduction in the splenic and hepatic parasitism was observed, suggesting that this triterpene potentialized AmB activity, mainly the doses of 0.2 and 1.0 mg/kg. In the monotherapy regimen, performed with AmB, reduction in the parasitism in the both spleen and liver was detected only in the highest dose studied (5.0 mg/kg); this means that AmB becomes active in vivo when animals receive a total dose of 18.75 mg, that according to previous works, this amount of drug, as well as lower ones, induced severe morphological and biochemical changes in the kidney of the experimental animals [21,31]. In contrast, non-therapeutic doses of AmB became therapeutic when combined with the triterpene UA, and it can be considered as an advantage, since combination carried out with low doses of AmB and UA eliminated an elevated number of viable parasites and also may induce less side-effects to the host. Additionally, it was verified that 1.0 mg/kg of UA administered alone reduced the number of viable parasites in the spleen and liver, as previously mentioned; in contrast, when 5.0 mg/kg of AmB was combined with UA, fewer parasites were eliminated in comparison to the respective monotherapy schemes, suggesting an antagonistic effect of this combination. However, it is still important to note that the association eliminated tissue amastigotes in the spleen and liver, whereas a low dose of AmB administered as monotherapy did not. These results are in agreement with those mentioned by Van Griensven [10], where the effect of two drugs administered in association may be smaller than the effect of each drug alone, suggesting that an antagonistic effect in the in vivo association [10] may occur. In spite of that, it is still important to highlight that the treatment of animals with the combination of AmB and UA could be considered advantageous, since in this experimental situation, only 0.2 mg/kg of AmB (total accumulated amount of AmB 0.75 mg) was needed to decrease the parasitism by 76% in the spleen, and thus, this dose of AmB may induce lesser side-effects than 5.0 mg/kg (total accumulated amount of AmB 18.75 mg); this means that each animal will receive 25 times less AmB when UA is associated.

In experimental cutaneous leishmaniasis, it was verified that Glu, given as monotherapy, had a dose-dependent therapeutic activity on infected BALB/c mice, in which 2.0 mg/kg had no impact on the parasitism, 10.0 mg/kg showed mild activity, and 50.0 mg/kg drastically reduced the skin parasitism by 96.3% and improved the skin lesion morphology. Furthermore, UA administered alone also reduced parasitism by 96.2%. In addition to the monotherapy scheme, it was observed that UA given as a coadjutant drug made the non-therapeutic dose of Glu (2.0 mg/kg) into an active dose, decreasing the number of viable parasites in the skin by 97.6% and improving the morphology of lesions in the base of the tail in infected BALB/c mice. A similar effect was observed in animals treated with 10.0 mg/kg of Glu, where monotherapy reduce parasitism by 73.6% while associated treatment decreased the skin parasitism by 99.8% and a trend to heal the skin lesions was observed. The treatment with 50.0 mg/kg of Glu alone or combined with UA significantly reduced the skin parasitism and although not statistically significant, the associated treatment tends to be more effective (99.4% of reduction) than monotherapy (96.3%). Altogether, these results strongly suggest an additive or synergistic effect of both drugs during the treatment in experimental cutaneous leishmaniasis. Previous studies have also observed similar behavior when miltefosine, a drug used in the treatment of leishmaniasis, was combined with the experimental drug tamoxifen. In this case, combined drugs were highly effective at controlling parasites in the experimental cutaneous leishmaniasis when compared to monotherapy [32], suggesting that combination of active drugs with different targets on *Leishmania* parasites can be a more effective treatment than monotherapy.

In fact, Glu became more effective at treating experimental animals with cutaneous leishmaniasis when associated with UA. For example, it was observed an almost total reduction in the tissue parasitism (97.5%) with a total dose of 0.75 mg Glu given over 15 days in association with UA. However, in monotherapy, a similar reduction in the number of viable parasites in the skin was found only in animals treated with a total amount of 18.75 mg Glu; thus, by adding UA to the treatment, 25 times lesser Glu was required to achieve a similar rate of effectiveness. In fact, such reduction in the amount of drug injected into the hosts can decrease all local and systemic side-effects induced by antimonials [33,34,35].

In respect to the immunological response, it has been observed that in visceral leishmaniasis, parasites downregulate IFN-γ, a Th1 related cytokine, and in contrast upregulate IL-10, a T regulatory associated cytokine [36,37]. Thus, a low or absent level of circulating IFN-γ will not activate macrophage to a leishmanicidal state; additionally, IL-10 is able to inhibit macrophage activation, and is associated with parasite persistence in the host tissues [38]. In the present study, it was observed that the spleen of infected hamsters treated with UA (1.0 mg/kg), AmB (5.0 mg/kg), and the combination (UA plus 5.0 mg/kg) expressed significantly more IFN-γ in comparison to the infected control [39], suggesting that besides the leishmanicidal activity of both drugs [21,40]), the therapeutic activity observed was accounted for by the immunomodulatory activity. On the other hand, IL-10 levels did not reduce after treatment, and the maintenance of such a cytokine during infection may limit or even inhibit the activity of macrophages [38,41] in the spleen, explaining, at least in part, the persistence of parasites in animals treated with AmB alone or when associated with UA.

In experimental cutaneous leishmaniasis, resistance is associated with IFN-γ production and susceptibility to IL-4 [42,43]. IL-4 cytokine is responsible for maturing B cells, which, in turn produce antibodies, that in leishmaniasis are ineffective, since parasites are hidden in the intracellular environment [44]. In monotherapy, only the groups treated with 1.0 mg/kg of UA or 10.0 mg/kg of Glu increased IFN-γ production. In contrast, the animals treated with all doses of Glu plus UA showed augmentation of this cytokine, suggesting that UA was able to modulate the immune system of treated animals, and such activity may be related to macrophage activation [22]. Previously, it was demonstrated that an UA-enriched fraction, purified from the leaves of *Baccharis uncinella*, was efficient at eliminating tissue amastigotes in experimental CL and these animals produced elevated amounts of IFN-γ [26], reinforcing that UA has a modulatory activity on the immune system. Besides, it is still important to note that UA has a direct effect on *Leishmania* sp. [21,22], and in vivo parasite elimination can be the sum of both leishmanicidal and immunomodulatory activities. Additionally, it is important to note that from an immunological point of view, UA and Glu showed an additive or potentializing effect on the Th-1 immune response, since groups treated with combined drugs did not produce lower levels of IFN-γ than the respective monotherapy regimen, and importantly all doses in combined therapy improved the overall aspect of macroscopic lesions than the respective monotherapy control, as observed in Figure 3E,G,I. In spite of IFN-γ increasing in the majority of the treated groups, animals treated with Glu alone or combined still produced IL-4, that at least in part, may reduce the bioactivity of IFN-γ, avoiding a sterile cure. In spite of that, in all experimental conditions, IFN-γ levels were higher than IL-4 levels, reinforcing that the therapeutic activity of this combination may be related to the direct effects of Glu, UA, and immunomodulatory activity.

Taken together, the results presented herein suggest that UA combined with the conventional drugs AmB and Glu can potentialize their activities in leishmaniasis in comparison to monotherapy schemes, especially in experimental cutaneous leishmaniasis. Although in VL, AmB plus UA did not present additive effects, mainly in the highest dose, it may still be considered as a viable alternative for the treatment of VL, given that the association required lower amounts of AmB to reduce tissue parasitism, that in turn may create a less toxic treatment and consequently greater patient compliance and tolerance. In experimental cutaneous leishmaniasis, UA potentialized the activity of Glu, and possibly an additive or synergistic effect was obtained, since a robust therapeutic effect compared to the monotherapy was observed; additionally, Th1 immune response was higher in groups treated with combined therapy compared to monotherapy, which is an important factor to efficiently reduce tissue parasitism. Altogether, these data suggest that combined therapy may be an interesting strategy to increase the efficacy of classical drugs in the treatment of leishmaniasis.

## 4. Materials and Methods

### 4.1. Drugs

UA was purchased from Cayman, USA, injectable glucatime and deoxycholate amphotericin B drugs from Sanofi-Aventis (São Paulo; Brazil) and Cristália (São Paulo; Brazil), respectively.

### 4.2. Chemical Analysis of Ursolic Acid

^1^H (300 MHz) and ^13^C (75 MHz) Nuclear Magnetic Resonance (NMR) spectra were recorded on deuterochloroform (CDCl_3_), deuterated dimethyl sulfoxide (DMSO-d_6_) or deuterated methanol (CD_3_OD) (Merck) to UA using a Bruker DPX-300 spectrometer. Elemental analysis of UA was obtained in a Perkin-Elmer Elemental Analyzer model 2400 CHN.

### 4.3. Experimental Animals and Ethical Considerations

Male Golden hamsters (*Mesocricetus auratus*), eight weeks old, were obtained from Anilab (Paulínia, São Paulo-Brazil) and male BALB/c mice, five weeks old, from the Medical School of the University of São Paulo, Brazil. This study was carried out in strict accordance with the recommendations detailed in the Guide for the Care and Use of Laboratory Animals of the Brazilian National Council of Animal Experimentation (http://www.cobea.org.br). The protocol was approved by the Ethics Committee of Animal Experiments of the Institutional Committee of Animal Care and Use at the Medical School of São Paulo University (CEUA 056/16). Hamsters and mice were housed according to the standards of the Committee of Animal Welfare, and allowed access to food and water ad libitum throughout the study under a 12 h light cycle. The animals were anesthetized with intraperitoneal sodium thiopental.

### 4.4. Parasites

*L. (L.) infantum* (MHOM/BR/72/46) and *L. (L.) amazonensis* (MHOM/BR/73/M2269) were kindly provided by Prof. Dr. Fernando Tobias Silveira from the cryobank of the “Leishmaniasis Laboratory Prof. Dr. Ralph Laison”, Department of Parasitology, Ministry of Health, Evandro Chagas Institute (Belém, Pará-Brazil). They were identified using monoclonal antibodies and isoenzyme electrophoretic profiles at the Leishmaniasis Laboratory of the Evandro Chagas Institute. Parasites were maintained in Schneider’s medium supplemented with 10% heat-inactivated fetal bovine serum, 10 μg/mL streptomycin, and 10 IU/mL ampicillin (ThermoFisher) (S10). Parasites in late log stage were used for all the experiments.

### 4.5. Evaluation of the Therapeutic Potential of Drug Associations in Visceral and Cutaneous Leishmaniasis

Forty golden hamsters (eight weeks old, weight ~ 250 g) were inoculated intraperitoneally with 100 µL of the parasite suspension (2 × 10^7^ *L. (L.) infantum* promastigotes). Five healthy control group was injected with PBS alone. After 45 days of infection, groups were arranged as follows: groups 1; 2 and 3 were treated with 1.0 mg/kg UA plus 0.2, 1.0 or 5.0 mg/kg of AmB, respectively. Group 4; 5 and 6 were treated exclusively with AmB, at 0.2; 1.0 and 5.0 mg/kg respectively; group 7 was treated with 1.0 mg/kg of UA; group 8 was the infected control group injected with vehicle solution and group 9 was untreated and uninfected animals (healthy control), injected with vehicle solution. In experimental visceral leishmaniasis, amphotericin B was chosen as the standard treatment, because in our model, glucantime showed low efficacy (data not show). A solution of associated UA + AmB was administered by the intraperitoneal route for 15 consecutive days. One week after the last injection, animals were sacrificed in CO_2_ chamber, and the spleen and liver were collected to determine splenic and hepatic parasitism. Each group was composed of at least four hamsters and the experiment was repeated three times, independently. UA was solubilized in DMSO and further PBS (never exceeding 1% DMSO); and AmB was solubilized in sterile water. The highest doses of 1.0 and 5.0 mg/kg from AmB and UA, respectively, were selected based on previously published works [21,45,46].

Forty BALB/c mice (four weeks old; weight ~ 25 g) were inoculated at the base of the tail with 2 × 10^7^ promastigote forms of *L. amazonensis* in stationary phase of growth (final volume 25 µL) as described in previous published works [9,46,47,48,49]. Five healthy control group was injected only with physiological solution. After 45 days of infection, the groups were arranged as follows: groups 1; 2 and 3 were treated with 1.0 mg/kg of UA plus 2.0, 10.0 or 50.0 mg/kg of glucantime, respectively; groups 4; 5 and 6 were treated exclusively with Glu (2.0, 10.0 and 50.0 mg/kg); group 7 was treated with 1.0 mg/kg of UA, and group 8 was the infected control group injected with vehicle solution while group 9 was untreated and uninfected animals, injected with vehicle solution (healthy control). All the treatments were given with a single daily dose of 20 µL, under intralesional route. The highest doses of 50.0 mg/kg from Glu was selected based on previously published works [47,50,51]. Treatment outcome was determined by skin parasitism that was analyzed one week after the end of treatment. Before, during, or after experimental treatments deaths were not recorded. 

### 4.6. Determination of Parasite Load

The parasite load was determined by limiting dilution assay [21,52]. In VL, parasitism was quantified in the spleen and liver, while in cutaneous leishmaniasis, it was analyzed in the skin at the point of parasite inoculation. Briefly, the skin, spleen, and liver were individually weighed, homogenized in S10, and then diluted 1:2000 (skin) or 1:500 (spleen and liver). An initial homogenized suspension was placed into the first well (200 μL) and serial dilutions (1:4) were distributed in a 96-multiwell plate (Nunc, Germany) and subjected to 12 serial dilutions with four replicate wells. After 10 days at 25 °C, each well was examined by optical microscopy and the final titer was set as the highest dilution for which the well contained at least one parasite. The viable parasitic load per gram of homogenized organ was calculated as follows: (reciprocal titer of the last positive well per total volume of homogenized tissue x dilution factor) divided by the weight (gram) of the homogenized tissue. The parasite load was expressed as the number of parasites per gram of homogenized organ. 

### 4.7. Analysis of Cellular Immune Response in VL and CL

RNA from hamster spleen fragments (~10 mg) was extracted using the commercial RNeasy Mini Kit (Qiagen, Hilden, Germany) according to the manufacturer’s protocol. cDNA was synthesized with the SuperScript^®^VILO™ cDNA Synthesis Kit (Life Technologies). Amplification conditions consisted of an initial denaturation phase at 95 °C for 10 min, followed by 40 amplification cycles consisting of 95 °C for 15 s, 61 °C for 90s, and 72 °C for 30 s, using a thermocycler (Eppendorf, Hamburg, Germany). Prior to quantification, the efficiency of each qPCR reaction was verified using cDNA from spleens of healthy animal; it was always above 95%. Expression levels of genes of interest were normalized to β-actin (endogenous control). qPCR reaction was carried out using the GoTaq*^®^*1-Step RT-qPCR System (Promega Corporation, Madison, WI, USA) and 75 nM of specific primer pairs. The primer sequences were as follows (5′ to 3′): IFN-γ forward: GACAACCAGGCCATCC and reverse: CAAAACAGCACCGACT; IL-10 forward: TGGACAACATACTACTCACTG and reverse: GATGTCAAATTCATTCATGGC; b-actin forward: TCCTGTGGCATCCACGAAACTACA and reverse: ACAGCACTGTGTTGGCATAGAGGT [53]. The amplification conditions consisted of 95 °C for 10 min followed by 40 cycles of 95 °C for 15 s, 57 °C for 30 s, and 72 °C for 30 s. Each sample was analyzed at least in duplicate. Results were expressed as relative gene expression to samples from infected hamsters. PCR products were electrophoresed on 2% agarose gels to confirm amplification of products with the correct size.

The determination of IL-4 and IFN-γ cytokines in BALB/c was analyzed in the supernatants of lymph node cultures stimulated with the whole antigen produced with *L. (L.) amazonensis* promastigotes (AgT). Lymph node cells from treated animals (5 × 10^5^ cells/well) were cultured in RPMI 1640 medium (R10 medium) supplemented with 10% fetal bovine serum, 1% L-glutamine, 10 μg/mL streptomycin, and 10 IU/mL ampicillin (ThermoFisher) under stimulation with 50 μg of whole antigen of *L. amazonensis* or 10 μg of concanavalin A as a positive control; negative controls were incubated only with R10 medium. After 72 h, the supernatants of the different groups were collected, and the amounts of IL-4 and IFN-γ (BD, Franklin Lakes, NJ, USA) were quantified by sandwich enzyme-linked immunosorbent assay (ELISA) in accordance with the manufacturer’s recommendations.

### 4.8. Statistical Analysis

All experiments were repeated at least three times, and the results were expressed by the arithmetic mean ± standard deviation. Statistical analyses were performed using GraphPad Prism 5.0, and the statistical test Kolmogorov-Smirnov was used, followed by one-way ANOVA. Differences were considered statistically significant at a 5% significance level (*p* < 0.05).
